# The p.G534E variant of *HABP2* is not associated with sporadic papillary thyroid carcinoma in a Polish population

**DOI:** 10.18632/oncotarget.16870

**Published:** 2017-04-06

**Authors:** Artur Kowalik, Danuta Gąsior-Perczak, Martyna Gromek, Monika Siołek, Agnieszka Walczyk, Iwona Pałyga, Małgorzata Chłopek, Janusz Kopczyński, Ryszard Mężyk, Aldona Kowalska, Stanisław Góźdź

**Affiliations:** ^1^ Department of Molecular Diagnostics, Holycross Cancer Centre, Kielce, Poland; ^2^ Department of Surgery and Surgical Nursing with The Scientific Research Laboratory, The Faculty of Health Sciences of The Jan Kochanowski University, Kielce, Poland; ^3^ Endocrinology Clinic, Holycross Cancer Centre, Kielce, Poland; ^4^ Genetic Clinic, Holycross Cancer Centre, Kielce, Poland; ^5^ Department of Surgical Pathology, Holycross Cancer Centre, Kielce, Poland; ^6^ Cancer Epidemiology, Holycross Cancer Centre, Kielce, Poland; ^7^ Oncology Clinic, Holycross Cancer Centre, Kielce, Poland; ^8^ The Faculty of Health Sciences, Jan Kochanowski University, Kielce, Poland

**Keywords:** p.G534E, sporadic papillary thyroid carcinoma, *HABP2*, thyroid cancer, non-medullary thyroid cancer

## Abstract

Thyroid cancer is one of the most frequently diagnosed cancers of the endocrine system. There are no known genetic risk factors for non-medullary thyroid cancer, other than a small number of hereditary syndromes; however, approximately 5% of non-medullary thyroid cancer, designated familial non-medullary thyroid cancer, exhibits heritability. The p.G534E (c.1601G>A) variant of *HABP2* was recently reported as a risk factor for familial non-medullary thyroid cancer, including papillary thyroid carcinoma. We analyzed the incidence of the c.1601G>A variant of *HABP2* in a Polish population consisting of 326 cases of papillary thyroid carcinoma and 400 control individuals by DNA genotyping, performed by Sanger sequencing. The c.1601G>A variant was detected in 3.7% of sporadic papillary thyroid carcinoma cases and 4.7% of healthy controls, and we did not detect an association between this variant and sporadic papillary thyroid carcinoma risk (OR = 0.71, 95% CI: 0.33–1.51; *p* = 0.3758). Additionally, no significant associations were identified between clinical and pathological disease features, response to primary treatment, and clinical status at the end of the observation, and *HABP2* c.1601G>A genotype. In conclusion, the p.G534E variant of *HABP2* is not associated with sporadic papillary thyroid carcinoma risk in the Polish population.

## INTRODUCTION

Thyroid cancer (TC) is one of the most frequently diagnosed endocrine malignancies worldwide [1] and its incidence is rising in Poland and elsewhere [2]. Other than hereditary syndromes (Cowden Syndrome, Carney Complex, Werner Syndrome), there are no known genetic risk factors for the development of non-medullary thyroid cancer (NMTC); however, approximately 5% of NMTC appears to exhibit heritability, and is classified as familial non-medullary thyroid cancer (FNMTC) [3]. Among NMTC cases, 85% are papillary thyroid cancer (PTC) [4]. Recently, Gara et al. described a multigenerational family cosegregating the c.1601G>A variant of *HABP2* (rs7080536; p.G534E), which sparked hope of a new genetic marker that could be useful for TC patient stratification and clinical management. Their investigations also demonstrated a role for *HABP2* as a tumor suppressor, suggesting that the identified variant is pathogenic [5]. Since then, several papers have reported an inability to confirm the importance of the p.G534E variant of *HABP2* in the development of FNMTC [7–11], with only one report supportive of the association [6]. Although research has been conducted using data from European populations (English and Spanish), there is a lack of information about the prevalence of this variant in PTC patient populations in Eastern Europe [12, 13]. Therefore, we assessed the prevalence of the c.1601G>A variant of *HABP2* in patients with sporadic PTC and a control Polish population, characterized by high homogeneity.

## RESULTS

Using Sanger sequencing, we detected the c.1601G>A variant of *HABP2* in 12/326 (3.7%) sporadic PTC patients; 11 (3.4%) carried the heterozygous (GA) and one (0.3%) the homozygous (AA) genotype at this locus. In the control group, the heterozygous GA genotype was detected in 19/400 (4.75%) patients with no homozygotes detected (Table 1). Statistical analysis indicated no significant difference in the incidence of the variant between the PTC and control populations (Fisher's exact test, *p* = 0.580902), and we did not detect an association between the c.1601G>A variant of *HABP2* and risk of sporadic PTC by logistic regression analysis (OR = 0.71, 95% CI: 0.33–1.51, *p* = 0.3758).

**Table 1:** Details of PTC patients and controls

	PTC patients	Controls	Test	Significance
**Number of samples**	326	400	Reference	
**Number with genotype, AA**	1	0	NA	NA
**Number with genotype, GA**	11	19	Logistic regression	OR (95% CI), 0.71 (0.33–1.51); *p* = 0.3758
**Percentage with genotypes GA+AA**	3.7	4.7	Fisher's exact test (vs. GG genotype)	*p* = 0.580902
**Mean age (SD)**	53.5 (13.5)	53.6 (9.9)	Independent Sample t-test	*p* = 0.8461
**F:M**	296:30 (90.8%:9.2%)	316:84 (70%:30%)	χ^2^	*p* < 0.0001

F, female; M, male; SD, standard deviation; χ^2^, Chi-squared test, NA, not applicable.

In addition, we assessed the correlation between clinical and pathological disease features, response to primary treatment, and clinical status at the end of the observation and detected *HABP2* genotypes (GG, GA, AA) in the PTC patient group. The results revealed no statistically significant differences (Table 2). In the group of sporadic PTC patients, only one patient (with a GG genotype at the *HABP2* variant) was classified as FNMTC according to the algorithm applied by our Outpatient Genetic Clinic (FNMTC was defined as the presence of at least three cases of PTC in a family). In addition, the homozygous AA genotype was detected in only one 63-year-old female patient with sporadic PTC.

**Table 2:** Evaluation of associations between detected *HABP2* genotypes (GG, GA, and AA) and clinical variables

Characteristic		Genotype			Statistical significance
		GG	AA	GA	
Gender	F/M	286/28	1/0	10/1	NS
Median tumor diameter (mm)		8	25	13	NS
Age (years)		50	62	37	NS
Tumor stage	T1	228	0	7	NS
	T2	19	0	1	
	T3	54	1	3	
	T4	13	0	0	
Lymph node involvement	N0	123	1	5	NS
	N1	28	0	0	
	Nx	163	0	6	
Metastasis	M0	313	1	11	NS
	M1	1	0	0	
Extra-thyroidal invasion	Yes	63	0	2	NS
	No	251	1	9	
Multifocality	Yes	261	1	8	NS
	No	53	0	3	
PTC type	Classical	276	1	7	NS
	Follicular	35	0	4	
	Oxyphilic	3	0	0	
Response to therapy	Excellent	280	1	10	NS
	Indeterminate	14	0	1	
	Biochemically incomplete	3	0	0	
	Structurally incomplete	17	0	0	
Follow-up (years)	Median (Min–Max)	7 (1–48)	6 (6–6)	5 (3–15)	NS
Status of final follow-up	No evidence of disease	295	1	10	NS
	Biochemically persistent disease	17	0	1	
	Persistent structural disease	1	0	0	
	Recurrence	1	0	0	
	Death	0	0	0	
	Total	314	1	11	

Associations between genotypes and clinical variables were assessed using *χ*^2^ and Kruskal-Wallis tests. NS, not statistically significant; F, female; M, male.

## DISCUSSION

The basis for the inherited genetic predisposition of FNMTC is largely unknown. Recently, Gara et al. published the results of a study indicating a relationship between the c.1601G>A (rs7080536; G534E) variant in the *HABP2* gene and PTC in a large multigenerational family [5]. All but one [6] subsequent report failed to confirm these results in analyses of dozens of families, including large families from different populations (Spain, USA, Australia, and the Middle East), that did not identify cosegregation of the c.1601G>A variant of *HABP2* with PTC [11, 13–15].

There is some controversy regarding the definition of FNMTC, which can be designated by as few as two cases of TC in the immediate family. Due to the high incidence of TC, it is possible for two cases of the disease to be a chance occurrence; hence diagnosis of FNMTC on the basis of only two cases may be incorrect [16, 17], while extension of the definition to require a minimum of three cases in a family improves specificity [18]. In the one report supportive of the *HABP2* association [6], two of four analyzed families included more than two cases of PTC (first- or second-degree relatives), while in the remaining FNMTC families there were only two cases of PTC, including one in a cousin (second-degree relative). In the present study, only one patient was from a FNMTC family, and we did not detect the c.1601G>A variant of *HABP2* in the proband.

Gara et al. [5] detected the p.G534E variant at a frequency of 4.7% (19 of 423) in data from multi-ethnic sporadic PTC patients in the Cancer Genome Atlas (TCGA), compared with 0.7% of those with unknown disease status. Immediately following their report, several groups were unable to repeat their findings; the reported frequencies of the p.G534E polymorphism in different NMTC populations (from China, the Middle East, Columbia, the UK, Australia, and the USA) vary from 0% to 7.8%. Similarly, the frequencies of the polymorphism in control groups from the same populations range from 0% to 9.3%. In all of these populations, no significant differences were identified in the frequency of the p.G534E variant of *HABP2* between NMTC and control groups [9, 11–15, 19]. In the present study of patients with sporadic PTC, the c.1601G>A variant of *HABP2* was detected in 3.7% of cases, while it was present in 4.75% of controls. Hence, we did not detect an association between the p.G534E variant of *HABP2* and sporadic PTC risk; nor was there any association of this variant with any clinical variables included in the study (Tables 1 and 2). Similarly, other published studies failed to detect associations of the variant with NMTC risk or other clinical variables [12, 19]. The reported absence of the p.G534E variant of *HABP2* in a Middle Eastern pediatric and juvenile NMTC population (5–26 years old) provides additional evidence that the variant is not associated with this disease [13, 15].

According to 1000 genomes project data (http://www.1000genomes.org/), the c.1601G>A variant of *HABP2* is present in 5.2% of Europeans. In the present study of a Polish population, the variant frequency was 4.75%, placing it in the middle of the range of values observed in other European populations (Finnish, 7.1%; British, 9.9%; Iberian, 2.8%; and Toscani (Italian), 2.8%).

In conclusion, based on our results, the p.G534E variant of *HABP2* is not associated with sporadic PTC and is not a risk factor for PTC development in a homogeneous Eastern European Polish population.

## MATERIALS AND METHODS

### Patients and controls

This study included 326 patients with sporadic PTC. Their mean age at diagnosis was 53.5 years (SD, 13.5 years), and one tenth of them were male (Table 1). A group of 400 healthy individuals (mean age, 53.6 years; SD, 9.9 years) was included in the study as a control cohort. There was no significant difference in age between cases and controls (*p* = 0.8461). All cases and controls were recruited at the Holycross Cancer Center in Kielce. All study participants provided written informed consent before participating in the research. All study procedures were and performed according to Declaration of Helsinki.

### Materials

Genomic DNA isolated from whole blood samples, obtained from all study participants, was used for genetic analyses. Material was extracted using a commercial DNA extraction kit (Genomic Micro AX BLOOD Gravity; A & A Biotechnology, Gdańsk, Poland), according to the manufacturer's instructions. DNA quality and purity were verified using a NanoDrop spectrophotometer (Thermo Fisher Scientific).

### Sanger sequencing

*HABP2* mutation analysis was performed by sequencing of PCR products. A target DNA region (181 bp) was amplified using KAPA SYBR Fast DNA Polymerase (KAPA BIOSYSTEMS) and the following oligonucleotide primer pair: forward 5′-TCTCTGGTTCACGAGGATGA-3′ and reverse 5′-GGCTTTGATCCAATTCAGGA-3′. The amplification conditions consisted of an initial denaturation at 95°C for 3 min, then 35 cycles of denaturation at 95°C for 30 s, annealing at 65°C for 30 s, and extension at 72°C for 20 s, followed by a final extension at 72°C for 2 min. PCR was performed using a Veriti Thermal Cycler (AppliedBiosystems). PCR products were separated by microchip electrophoresis, using MultiNA system (Shimadzu Corporation, SHIM-POL, Poland). Amplified material was purified using FastAP and Exonuclease I (Thermo Fisher Scientific), according to the manufacturer's protocol. Sequencing was carried out using the same forward primer that was used for PCR amplification (see above). The primer was diluted at a ratio of 8:42 μl in water. Other sequencing reagents were as follows: BigDye® Terminator v1.0 or v3.0 and 5X Sequencing Buffer (Thermo Fisher Scientific). The sequencing reaction was performed using the Veriti Thermal Cycler (Applied Biosystems) and the following conditions: 25 cycles at 96°C for 10 s, 50°C for 5 s, and 60°C for 1 min 45 s. The reactions were then purified using a BigDye XTerminator® Purification Kit (SAM Solution and XTerminator Solution; Thermo Fisher Scientific). Next, products were separated and analyzed using an ABI 3130 Genetic Analyzer (Applied Biosystems). The resulting DNA sequences were analyzed using BLAST (www.blast.ncbi.nlm.nih.gov).

### Statistical analyses

The mean age of cases and controls was compared using an Independent Samples t-test. To assess the predictive power of the genotypes for PTC risk (odds ratios, ORs), logistic regression was performed. Differences in the frequencies of genotypes between cases and controls were determined using a two-tailed Fisher's exact test. Associations between genotypes and clinical variables were assessed using *χ*^2^ and Kruskal-Wallis tests. Association between sexes and study groups was assessed usingχ^2^. Statistical significance was set at *p* < 0.05.

## Abbreviations

FNMTC: familial non-medullary thyroid cancer
NMTC: non-medullary thyroid cancer
PTC: papillary thyroid carcinoma
TC: thyroid cancer
